# Optical coherence tomography combined with convolutional neural networks can differentiate between intrahepatic cholangiocarcinoma and liver parenchyma ex vivo

**DOI:** 10.1007/s00432-023-04742-x

**Published:** 2023-04-12

**Authors:** Laura I. Wolff, Enno Hachgenei, Paul Goßmann, Mariia Druzenko, Maik Frye, Niels König, Robert H. Schmitt, Alexandros Chrysos, Katharina Jöchle, Daniel Truhn, Jakob Nikolas Kather, Andreas Lambertz, Nadine T. Gaisa, Danny Jonigk, Tom F. Ulmer, Ulf P. Neumann, Sven A. Lang, Iakovos Amygdalos

**Affiliations:** 1 Department of General, Visceral and Transplantation Surgery, University Hospital RWTH Aachen, Aachen, Germany; 2 Department of Production Metrology, Fraunhofer Institute for Production Technology IPT, Aachen, Germany; 3 Department of Production Quality, Fraunhofer Institute for Production Technology IPT, Aachen, Germany; 4 Laboratory for Machine Tools and Production Engineering (WZL), RWTH Aachen University, Aachen, Germany; 5 Department of Diagnostic and Interventional Radiology, University Hospital RWTH Aachen, Aachen, Germany; 6 Department of Internal Medicine III, University Hospital RWTH Aachen, Aachen, Germany; 7 Else Kroener Fresenius Center for Digital Health, Medical Faculty Carl Gustav, Carus Technical University Dresden, Dresden, Germany; 8 Institute for Pathology, University Hospital RWTH Aachen, Aachen, Germany; 9 German Center of Lungs Research (DZL, BREATH), Gießen, Germany

**Keywords:** Optical coherence tomography, Intrahepatic bile ducts, Computer neural networks, Machine learning, Cholangiocarcinoma, Deep learning

## Abstract

**Purpose:**

Surgical resection with complete tumor excision (R0) provides the best chance of long-term survival for patients with intrahepatic cholangiocarcinoma (iCCA). A non-invasive imaging technology, which could provide quick intraoperative assessment of resection margins, as an adjunct to histological examination, is optical coherence tomography (OCT). In this study, we investigated the ability of OCT combined with convolutional neural networks (CNN), to differentiate iCCA from normal liver parenchyma ex vivo.

**Methods:**

Consecutive adult patients undergoing elective liver resections for iCCA between June 2020 and April 2021 (*n* = 11) were included in this study. Areas of interest from resection specimens were scanned ex vivo, before formalin fixation, using a table-top OCT device at 1310 nm wavelength. Scanned areas were marked and histologically examined, providing a diagnosis for each scan. An Xception CNN was trained, validated, and tested in matching OCT scans to their corresponding histological diagnoses, through a 5 × 5 stratified cross-validation process.

**Results:**

Twenty-four three-dimensional scans (corresponding to approx. 85,603 individual) from ten patients were included in the analysis. In 5 × 5 cross-validation, the model achieved a mean F1-score, sensitivity, and specificity of 0.94, 0.94, and 0.93, respectively.

**Conclusion:**

Optical coherence tomography combined with CNN can differentiate iCCA from liver parenchyma ex vivo. Further studies are necessary to expand on these results and lead to innovative in vivo OCT applications, such as intraoperative or endoscopic scanning.

**Supplementary Information:**

The online version contains supplementary material available at 10.1007/s00432-023-04742-x.

## Background

Liver cancer is the sixth most common and third deadliest cancer worldwide (Sung et al. 2021). It includes hepatocellular carcinoma (HCC), which comprises 75–85% of cases, intrahepatic cholangiocarcinoma (iCCA), accounting for 10–15%, and other rarer malignancies (Sung et al. 2021). Only 12–40% of patients diagnosed with iCCA are eligible for surgical therapy (Mazzaferro et al. 2020), although radical surgical removal of all tumor tissue (R0) is currently the only curative treatment for iCCA without distant metastasis (Mazzaferro et al. 2020; Voesch et al. 2022). This fact underlines the importance of improving diagnostics to detect liver cancer earlier and to accurately determine resection radicality intraoperatively. The latter substantially relies on frozen section analysis, which can generally differentiate malignant from benign liver lesions with high sensitivity and specificity (up to 96.9% and 99.1%, respectively) (Rakha et al. 2006). Nevertheless, frozen section analysis is time-consuming (Nguyen et al. 2009; Zhang et al. 2017), leading to longer surgeries, which are associated with an increased risk of complications and costs (Nguyen et al. 2009; Cheng et al. 2018).

A non-invasive imaging technology, with the potential to address these challenges, is optical coherence tomography (OCT). Based on low-coherence interferometry, OCT produces real-time, high-resolution cross-sectional images at a depth of 1–3 mm, with axial and lateral resolutions of 1–20 μm, respectively (Garcia-Allende et al. 2011; Samel and Mashimo 2019; Zhu et al. 2020; Kufcsak et al. 2021; Amygdalos et al. 2022a). It has been previously shown that the combination of OCT with advanced processing modalities, such as machine learning (ML), can lead to high diagnostic accuracy (Aggarwal et al. 2021, Saratxaga, Bote et al. 2021, Amygdalos et al. 2022a). Machine learning is an artificial intelligence (AI) technique, where machines are trained to autonomously perform tasks, using computational methods (Amygdalos et al. 2022b). A subtype called deep learning (DL) utilizes neural networks, which are ML models consisting of connected layers. Here, each layer’s output serves as the input for the next (Goodfellow et al. 2016; Chollet 2017). In convolutional neural networks (CNN), convolutional layers apply multiple filters on the input to learn features in images (LeCun et al. 2015; Goodfellow et al. 2016; Chollet 2017). CNN extract features from images without requiring human performance (LeCun et al. 2015) and are increasingly used for image processing, including OCT. For example, in a previous study, we demonstrated a high sensitivity and specificity in the differentiation of colorectal liver metastases (CRLM) from healthy liver parenchyma using OCT combined with CNN (Amygdalos et al. 2022a). In several studies, CNN analyses were performed on OCT images, for example, to classify different types of retinal diseases (Alqudah 2020), to differentiate colorectal cancer from normal tissue (Zeng et al. 2020) and to investigate breast tumor margins (Mojahed et al. 2020). The combination of OCT with CNN or other ML modalities could provide significant clinical benefits. For example, intraoperative OCT could potentially control surgical margins faster than frozen section examination, helping to define resection planes and reducing operating time and cost (Amygdalos et al. 2022a). Moreover, endoscopic applications could lead to earlier and more accurate diagnosis of cholangiocarcinoma and other pathologies.

This study investigates the ability of spectral domain OCT (SD-OCT) combined with CNN to differentiate iCCA from normal liver parenchyma ex vivo.

## Methods

### Patient cohort and inclusion criteria

Consecutive adult patients undergoing elective liver resections for iCCA at the University Hospital RWTH Aachen (UH-RWTH) between June 2020 and April 2021 were included in this study. Patients undergoing emergency operations were excluded, as were those unable or unwilling to provide informed consent.

### OCT imaging system

The same table-top SD-OCT device (Telesto™ V1, Thorlabs GmbH, Lübeck, Germany) was used in this study as previously described (Amygdalos et al. 2022a), operating at 1310 nm wavelength. One-dimensional axial scans (A-scans) were combined by sweeping the laser beam to create two-dimensional (B-scan) and three-dimensional (C-scan) images. Between June and August 2020, the previously described scanning format was used, namely an area of 3.0 mm × 3.0 mm × 2.5 mm and resolution of 1024 × 1024 × 512 pixels, with a pixel size of 2.93 µm in x- and y-direction and 4.97 µm in z-direction. In the period from October 2020 to April 2021, the field of view was changed to a “wide format” setting of 9.90 mm × 2.55 mm × 2.55 mm, to increase the number of features per image and to accommodate further questions outside the scope of this study, such as scanning of the tumor–parenchyma interface (incorporating two tissue types in the same image). The “wide format” scans had a resolution of 2048 × 512 × 512 pixels, with a pixel size of 4.83 µm in *x*, 4.98 µm in *y* and 4.97 µm in *z*-direction. Each C-scan contained 512 B-scans, which was fewer than our previous study, but the total number of pixels per scan remained the same.

### Specimen collection and scanning

**Fig. 1:**
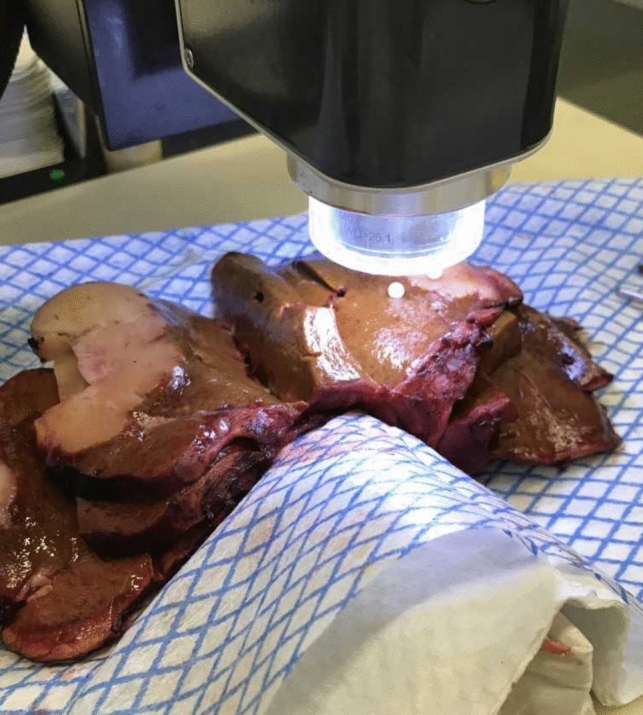
A typical OCT scanning orientation of a liver specimen with iCCA. A transition zone scan including healthy parenchyma and tumor has been marked with white pins

Specimen processing and data collection were performed as previously described (Garcia-Allende et al. 2011; Amygdalos et al. 2022a). Briefly, resected tissue specimens were immediately transferred to the histology department, where scanning of normal liver parenchyma and tumor was carried out, after completion of frozen section analysis. Liver specimens were cut into lamellae, allowing access to tumors within the parenchyma. Scanned sections were marked with pins. After completion of scanning, specimens were placed in formalin and marked sections were histologically examined and reported-on separately. A typical scanning orientation is shown in Fig. 1.

### OCT image processing and neural network analysis

**Fig. 2:**
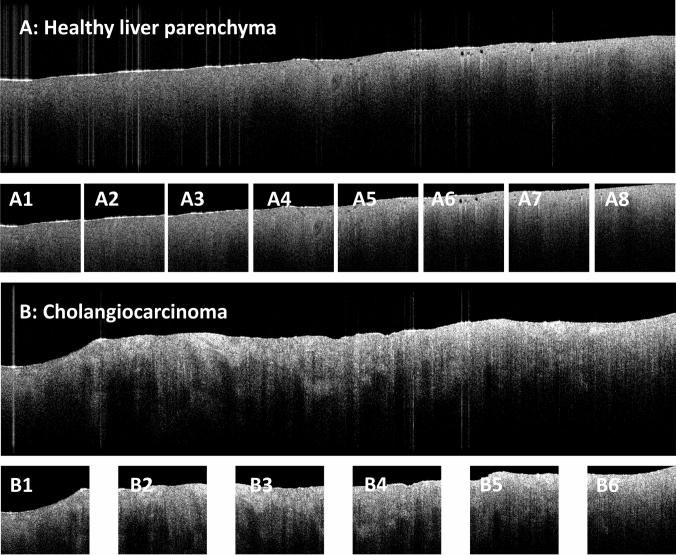
Two B-scans of the “wide format” of the same patient before and after image pre-processing, showing liver parenchyma (**A**) and tumor (**B**). The parenchyma scan was cropped into 8 images (A1–A8) at the end of image pre-processing, whereas the tumor scan was cropped into 6 images (B1–B6), all 299 × 299 pixels. The cropped images are then tagged as belonging to the same scan and fed into the convolutional neural network

All image processing and CNN analysis was carried out in the Anaconda environment (*Anaconda Software Distribution*, 2020. *Anaconda Documentation*. Anaconda Inc. Retrieved from https://docs.anaconda.com/), using the Python programming language (RRID:SCR_008394) (VanRossum 2010). An NVIDIA GeForce RTX 3060 Ti graphics processing unit with 8 GB graphics double data rate 6 synchronous dynamic random-access memory (Nvidia Corporation, California, United States) was used in this study. Preprocessing of OCT images, as well as training and validation of the CNN was along the lines of our previously described methodology (Amygdalos et al. 2022a). First, raw OCT images were reconstructed using metadata and intensity values in decibel, and then scaled to a 0–255 range for each C-scan. This involved analyzing the intensity range from top to bottom of the first, middle, and last B-scan, and scaling across the whole C-scan. Then, each C-scan was converted into a series of B-scans, which were pre-processed to correct artifacts, remove areas without useful information, and increase signal-to-noise ratio (SNR). The resulting images were then cropped into 299 × 299 pixel tiles for analysis with the Xception CNN (Chollet 2017). Multiple tiles were extracted from each B-scan, depending on its size after artifact correction. Quality control of the final images was performed before being included in the analysis and inadequate images were excluded, as previously described (Amygdalo﻿s et al. 2022a). “Wide format” parenchyma and tumor scans, before and after pre-processing, are shown in Fig. 2.

A pre-trained Xception CNN was sourced from Keras, an open-source application programming interface (API), and used to differentiate OCT scans according to their corresponding histological diagnoses. The original and modified architectures of Xception for OCT applications have been previously described (Chollet 2017; Saratxaga et al. 2021; Amygdalos et al. 2022a). Hyperparameters were set as follows: batch size 9, 10 epochs, learning rate 0.00001. Stratified k-fold cross-validation (CV) was used to split data 70:15:15 for training, validation, and testing, respectively. The CV process was carried out in 5 cycles (labeled A–E), each generating a different non-overlapping test data split. Within each cycle, 5 random, non-overlapping iterations of the training/validation split were produced. Individual C-scans were kept intact throughout all data-splitting and randomization processes, to prevent a falsely high accuracy (i.e., all images belonging to a C-scan were kept together within training/validation/test splits). The process produced 25 trained and tested versions of Xception (labeled A1 to E5). Results are reported separately for each version, as well as combined to provide mean values for the study.

A cross-entropy loss function was used to calculate prediction errors and construct confusion matrices for the CV process. These were used to calculate the sensitivity, specificity, F1-score and loss values. As previously described, the F1-score is the harmonic mean of positive predictive value and sensitivity, whereas loss is a metric of prediction accuracy, which describes how well the CNN is learning (Amygdalos et al. 2022a). Two sets of CV-analyses were performed, the first including scans of both formats (in the following referred to as “mixed format” analysis) and the second only using the “wide format” scans. This way, the effect of mixing formats could be examined.

Continuous data are presented as mean (standard deviation, SD) where applicable. The programming code has been uploaded to https://github.com/iamygdalos/OCT_CCC and the OCT data used in this study can be provided upon reasonable request to the corresponding author.

## Results

### Specimen statistics

A total of 11 patients underwent liver resection for iCCA at the UH-RWTH during the study period, from which 17 tumor areas and 16 healthy areas were scanned. Four scans of normal liver parenchyma and one of tumor were excluded, because of persisting image artifacts. To compensate for the resulting deficit of parenchyma scans, scans of normal parenchyma from 4 patients undergoing liver surgery for benign pathologies were added to the control group. This resulted in a balanced number of 32 scans (16 iCCA, 16 liver parenchyma) from 15 patients. The gender distribution of the iCCA patients was nearly balanced, with 45% men and 55% women. The mean age was 64 years.

### Xception CNN classification results

**Fig. 3:**
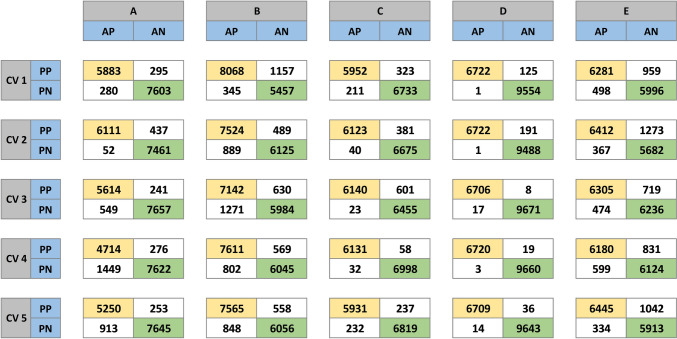
Confusion matrices of the “wide format” analysis for the 25 trained CNN models, derived from their predictions on the test set. The models are labeled A1–E5, according to the CV set and cycle they were trained and validated in; *AP* actual positive, *AN* actual negative, *PP* predicted positive, *PN* predicted negative

The CV analysis only including scans of the “wide format” contained 24 scans (12 of tumor and 12 of liver parenchyma) from 10 patients, comprising 85,603 images. Across all 25 trained and validated Xception CNN models making predictions on the test set, the mean F1-score, sensitivity, and specificity were 0.94 (0.04), 0.94 (0.06), and 0.93 (0.05), respectively. The F1-score ranged from 0.85 to 1.0, whereas sensitivity and specificity ranged from 0.76 to 1.0 and from 0.82 to 1.0, respectively. Detailed performance metrics derived from the predictions on the test set and confusion matrices of the “wide format” analysis are shown in Table 1 and Fig. 3. Overall analysis characteristics and results are shown in Table 2 and performance metrics of the mixed format analysis are presented in Supplementary Fig. 1 and Supplementary Table 1.

**Table 1:** Performance metrics of the “wide format” analysis for the 25 trained CNN models, derived from their predictions on the test set

CV cycle	Sensitivity	Specificity	PPV	NPV	Accuracy	F1-Score
A1	0.95	0.96	0.95	0.96	0.96	0.95
A2	0.99	0.94	0.93	0.99	0.97	0.96
A3	0.91	0.97	0.96	0.93	0.94	0.93
A4	0.76	0.97	0.94	0.84	0.88	0.85
A5	0.85	0.97	0.95	0.89	0.92	0.90
B1	0.96	0.83	0.87	0.94	0.90	0.91
B2	0.89	0.93	0.94	0.87	0.91	0.92
B3	0.85	0.90	0.92	0.82	0.87	0.88
B4	0.90	0.91	0.93	0.88	0.91	0.92
B5	0.90	0.92	0.93	0.88	0.91	0.91
C1	0.97	0.95	0.95	0.97	0.96	0.96
C2	0.99	0.95	0.94	0.99	0.97	0.97
C3	1.00	0.91	0.91	1.00	0.95	0.95
C4	0.99	0.99	0.99	1.00	0.99	0.99
C5	0.96	0.97	0.96	0.97	0.96	0.96
D1	1.00	0.99	0.98	1.00	0.99	0.99
D2	1.00	0.98	0.97	1.00	0.99	0.99
D3	1.00	1.00	1.00	1.00	1.00	1.00
D4	1.00	1.00	1.00	1.00	1.00	1.00
D5	1.00	1.00	0.99	1.00	1.00	1.00
E1	0.93	0.86	0.87	0.92	0.89	0.90
E2	0.95	0.82	0.83	0.94	0.88	0.89
E3	0.93	0.90	0.90	0.93	0.91	0.91
E4	0.91	0.88	0.88	0.91	0.90	0.90
E5	0.95	0.85	0.86	0.95	0.90	0.90
Mean	0.94	0.93	0.94	0.94	0.94	0.94
SD	0.06	0.05	0.05	0.05	0.04	0.04

*CV* cross-validation, *PPV* positive predictive value, *NPV* negative predictive value, *SD* standard deviation. The models are labeled A1–E5, according to the CV set and cycle in which they were trained and validated

**Table 2:** Analysis characteristics and results

Format	Mixed	Wide
No. of patients, total	15	10
No. of iCCA patients	11	9
No. of iCCA C-scans	17	13
No. of included iCCA C-scans	16	12
No. of images	113,642	85,603
Mean accuracy	0.89	0.94
Mean F1-score	0.88	0.94
Mean sensitivity	0.88	0.94
Mean specificity	0.90	0.93

## Discussion

This study demonstrates that OCT combined with the Xception CNN can accurately differentiate between iCCA and normal liver parenchyma ex vivo. In particular, the mean F1-score, sensitivity, and specificity of all trained Xception models were 0.94, 0.94, and 0.93, respectively.

In the field of OCT, CNN have been trained to detect retinal diseases (Alqudah 2020), colorectal cancer (Zeng et al. 2020), esophageal cancer (Fonollà et al. 2019), and breast tumors (Mojahed et al. 2020). Up till now, only a few studies have investigated OCT in liver tissues. Some ex vivo animal studies on liver tissue have focused on technical capabilities of OCT systems (Jain et al. 2011; Genina et al. 2012), whereas others have investigated the detection and grading of steatosis, inflammation, and fibrosis through OCT imaging (Wu et al. 2007; Mukherjee et al. 2021). In human livers, there have been proof-of-concept studies with formalin-fixed liver specimens (Zhu et al. 2015, 2020; Mu et al. 2019) and in vitro liver tissues (Zhou et al. 2015). These primarily focused on imaging, rather than diagnostic capabilities of various OCT systems, although Zhu et al. used a support vector machine (SVM) model to distinguish HCC from healthy liver parenchyma (Zhu et al. 2020).

This study, to the best of our knowledge, is only the second one to investigate human liver pathologies using a combination of OCT and CNN, after our previous work on CRLM (Amygdalos et al. 2022a). Once again, we employed a validated methodology, using a pre-trained Xception CNN and stratified CV, to produce 25 trained and validated models with consistently high F1-scores, highlighting the reproducibility of our results. Furthermore, we scanned fresh, whole resection specimens, using a non-destructive specimen-collection and -scanning methodology, which integrated well into the histopathological clinical process. This allowed us to examine a variety of tissue types, in scanning orientations applicable to real-life clinical scenarios, as well as build up a relatively large dataset in a limited time period. We also achieved similarly good results with the new “wide format” scans to those of our previous study (Amygdalos et al. 2022a), which demonstrates the adaptability of our image processing technique. However, better results were achieved when using only the “wide format” scans, compared to mixing the two formats, suggesting that a standardized format is required for future work.

Our study built on our previous proof-of-concept (Amygdalos et al. 2022a). While the methodology of tissue collection was the same as in our previous study, the scanning parameters changed. The new image dimensions resulted in a change in resolution and required adjustment of the programming code for pre-processing, in order for cropping to function properly. Moreover, changes to the CNN training hyperparameter batch size were made. Contrary to the first paper, the test set did not remain constant for all 25 CV runs, it rather changed for each CV cycle, and thus, there were 5 different test sets on which 5 trained and validated models were tested. While the argumentation in the first study was, that a constant test set would make results between models more comparable (Amygdalos et al. 2022a), here we demonstrated the constancy of good predictions even with a changing test set. Finally, the change in tumor type from CRLM to iCCA is a major novelty of this study. First, good results with a different tumor type are not guaranteed. Second, iCCA tumors are rarer than CRLM and this study is the only one investigating cholangiocarcinoma with OCT and machine learning, to the best of our knowledge.

Our study demonstrated potential for incorporation of OCT and CNN in future in vivo clinical applications. One possibility would be the quick intraoperative examination of liver resection margins, which would reduce the number of frozen sections and total operation time (Moller et al. 2021). Another could be the earlier and more accurate diagnosis of iCCA during endoscopic retrograde cholangiopancreatography (ERCP). The additional use of OCT alongside biliary brushing has already been shown to improve diagnostic sensitivity (Arvanitakis et al. 2009) and the addition of DL analysis could further improve on this. For this, a large dataset encompassing high-quality OCT data from various pathologies and healthy tissue must be acquired, followed by training of various CNN to determine the combination with the highest diagnostic accuracy for each clinical question. Furthermore, the CNN architecture could be modified to enable learning of more complex features, by increasing the number of hidden layers, tuning hyperparameters to improve learning rate, or processing whole volume data (C-scans) (Chollet 2017; Esteva et al. 2019; Saratxaga et al. 2021). Finally, the effect of wavelength and polarization-sensitivity of OCT devices could be further explored. A shorter wavelength increases resolution, but decreases penetration depth, and vice versa (Samel and Mashimo 2019). The ideal combination depends on the clinical application. For example, in examination of bile ducts with endoscopic OCT, penetration depth would be less important, as relevant pathologies would lie at the surface, so that a higher resolution would be more important. On the contrary, detection of diseased tissue below the surface, such as in scanning of resection margins, would require longer wavelengths, sacrificing resolution. Polarization-sensitive OCT detects changes in the polarization state of the scanning beam, increasing the image contrast and providing additional information about the tissue microstructure (Pircher et al. 2011). Studies on murine livers have demonstrated good visualization of collagen microstructures, such as fibrotic tissue and microvascular complexes (Mukherjee et al. 2021). This will be the aim of further studies, starting ex vivo and expanding into the in vivo domain.

Certain limitations of the present study must be considered, when interpreting our results. A larger data set, both in terms of patients included and images obtained from each patient, would strengthen our results, and decrease overfitting of the CNN during training and validation. A partial requirement for this would be an upgraded OCT system, which would allow for quicker and higher quality scanning, without delaying histological processing of specimens. Additionally, improvements in the scanning and pre-processing methodology would reduce the number of discarded C-scans. For example, an AI process for removing artifacts, trained on good-quality images, may be more effective than our current methodology. Furthermore, testing on external data would increase the validity of our results. However, there are little available data on OCT in liver tissues at the moment. Additionally, this study focused on liver parenchyma as the control tissue, whereas in real clinical settings, infiltration of portal fields, bile ducts, and connective tissues is a pertinent question. To investigate this, a larger dataset of OCT images is required, including tumor infiltration of these specific tissues, matched with detailed histological reporting of the scanned areas. Finally, transferability to the in vivo domain must still be investigated.

## Conclusions

In this study, we demonstrate that SD-OCT combined with Xception CNN can differentiate iCCA from liver parenchyma ex vivo with high overall sensitivity, specificity and F1-score. Further studies are necessary to prove the ability of OCT as a long-term complement to current diagnostics in clinical practice, especially in vivo.

## Supplementary Information

Below is the link to the electronic supplementary material.

Supplementary file1 (DOCX 1250 KB)

## Data Availability

The datasets analyzed during the current study are available from the corresponding author on reasonable request (iamygdalos@ukaachen.de).

## Abbreviations

AI: Artificial intelligence
AN: Actual negative
AP: Actual positive
API: Application programming interface
AUC: Area under the curve
CCA: Cholangiocarcinoma
CNN: Convolutional neural network
CRLM: Colorectal liver metastases
CT: Computed tomography
CV: Cross-validation
DL: Deep learning
ERCP: Endoscopic retrograde cholangiopancreatography
FF-OCT: Full-field optical coherence tomography
HCC: Hepatocellular carcinoma
iCCA: Intrahepatic cholangiocarcinoma
ML: Machine learning
MRI: Magnetic resonance imaging
NPV: Negative predictive value
OCT: Optical coherence tomography
PN: Predicted negative
PNG: Portable network graphics
PP: Predicted positive
PPV: Positive predictive value
RGB: Red–green–blue
SD: Standard deviation
SD-OCT: Spectral domain OCT
SNR: Signal-to-noise ratio
SVM: Support vector machine
UH-RWTH: University Hospital RWTH Aachen
