# Supplementary material for: Optical coherence tomography combined with convolutional neural networks can differentiate between intrahepatic cholangiocarcinoma and liver parenchyma ex vivo
Source: J Cancer Res Clin Oncol. 2023 Apr 12;149(10):7877–85. doi: 10.1007/s00432-023-04742-x (PMC10374764; doi:10.1007/s00432-023-04742-x)
Supplement: Supplementary file 1 — Supplementary file1 (DOCX 1250 KB) [file 432_2023_4742_MOESM1_ESM.docx]

Supplementary Material

Optical coherence tomography combined with convolutional neural networks can differentiate between intrahepatic cholangiocarcinoma and liver parenchyma *ex vivo*.

Supplementary Figure Legends and Tables

**Supplementary Figure 1.** Confusion matrices of the mixed format analysis for the 25 trained CNN models, derived from their predictions on the test set. The models are labelled A1-E5, according to the CV set and cycle they were trained and validated in; AP actual positive; AN actual negative; PP predicted positive; PN predicted negative.


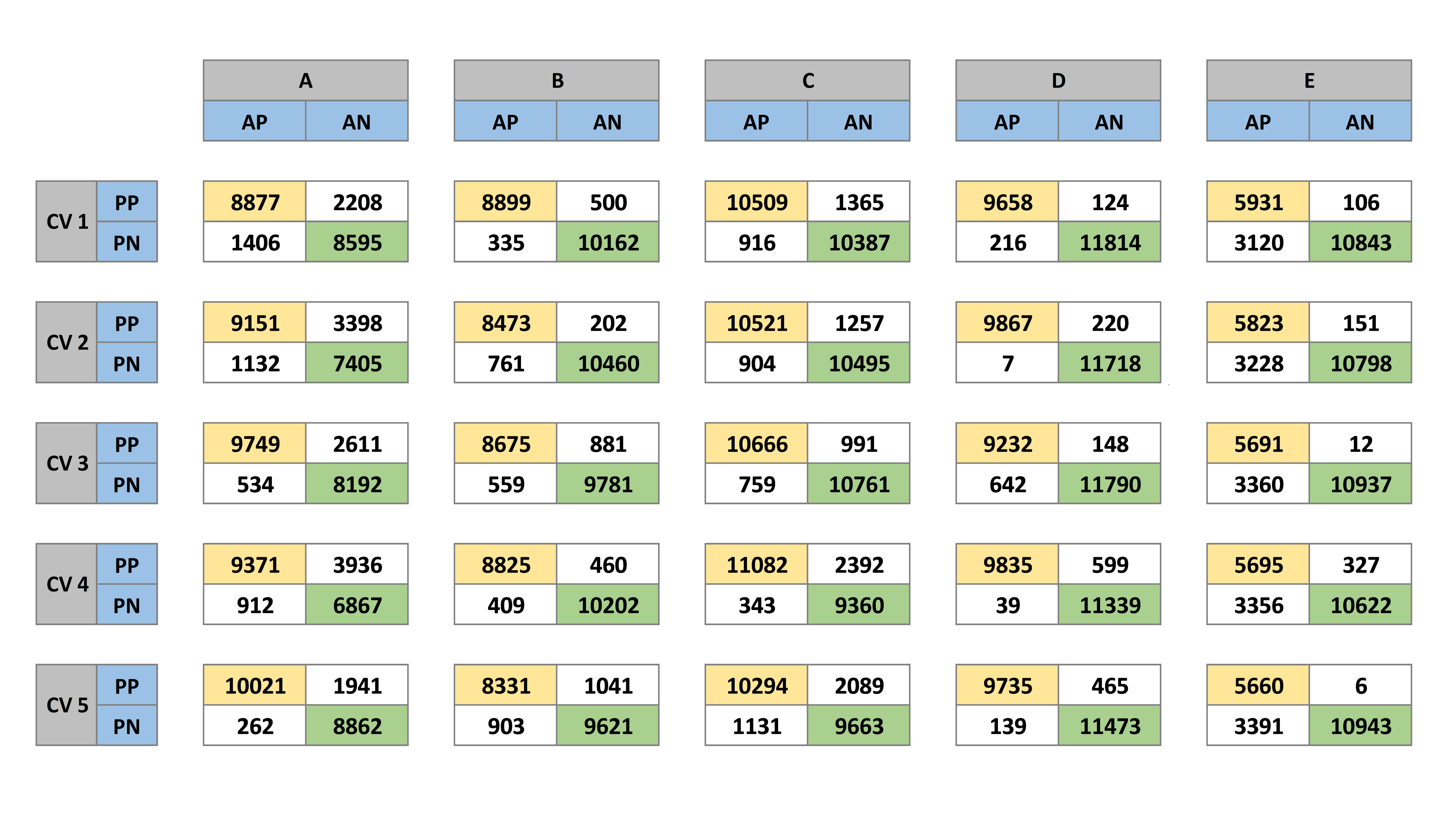


**Supplementary Table 1.** Performance metrics of the mixed format analysis for the 25 trained CNN models, derived from their predictions on the test set.

| CV cycle | Sensitivity | Specificity | PPV | NPV | Accuracy | F1-Score |
| --- | --- | --- | --- | --- | --- | --- |
| A1 | 0.86 | 0.80 | 0.80 | 0.86 | 0.83 | 0.83 |
| A2 | 0.89 | 0.69 | 0.73 | 0.87 | 0.79 | 0.80 |
| A3 | 0.95 | 0.76 | 0.79 | 0.94 | 0.85 | 0.86 |
| A4 | 0.91 | 0.64 | 0.70 | 0.88 | 0.77 | 0.79 |
| A5 | 0.97 | 0.82 | 0.84 | 0.97 | 0.90 | 0.90 |
| B1 | 0.96 | 0.95 | 0.95 | 0.97 | 0.96 | 0.96 |
| B2 | 0.92 | 0.98 | 0.98 | 0.93 | 0.95 | 0.95 |
| B3 | 0.94 | 0.92 | 0.91 | 0.95 | 0.93 | 0.92 |
| B4 | 0.96 | 0.96 | 0.95 | 0.96 | 0.96 | 0.95 |
| B5 | 0.90 | 0.90 | 0.89 | 0.91 | 0.90 | 0.90 |
| C1 | 0.92 | 0.88 | 0.89 | 0.92 | 0.90 | 0.90 |
| C2 | 0.92 | 0.89 | 0.89 | 0.92 | 0.91 | 0.91 |
| C3 | 0.93 | 0.92 | 0.91 | 0.93 | 0.92 | 0.92 |
| C4 | 0.97 | 0.80 | 0.82 | 0.96 | 0.88 | 0.89 |
| C5 | 0.90 | 0.82 | 0.83 | 0.90 | 0.86 | 0.86 |
| D1 | 0.98 | 0.99 | 0.99 | 0.98 | 0.98 | 0.98 |
| D2 | 1.00 | 0.98 | 0.98 | 1.00 | 0.99 | 0.99 |
| D3 | 0.93 | 0.99 | 0.98 | 0.95 | 0.96 | 0.96 |
| D4 | 1.00 | 0.95 | 0.94 | 1.00 | 0.97 | 0.97 |
| D5 | 0.99 | 0.96 | 0.95 | 0.99 | 0.97 | 0.97 |
| E1 | 0.66 | 0.99 | 0.98 | 0.78 | 0.84 | 0.79 |
| E2 | 0.64 | 0.99 | 0.97 | 0.77 | 0.83 | 0.78 |
| E3 | 0.63 | 1.00 | 1.00 | 0.76 | 0.83 | 0.77 |
| E4 | 0.63 | 0.97 | 0.95 | 0.76 | 0.82 | 0.76 |
| E5 | 0.63 | 1.00 | 1.00 | 0.76 | 0.83 | 0.77 |
| Mean | **0.88** | **0.90** | **0.90** | **0.90** | **0.89** | **0.88** |
| SD | **0.13** | **0.10** | **0.09** | **0.08** | **0.07** | **0.08** |

Legend: CV: cross-validation; PPV: positive predictive value; NPV: negative predictive value; SD: standard deviation. The models are labelled A1-E5, according to the CV set and cycle in which they were trained and validated.
